# Case Report: Tuberculosis IRIS: a mediastinal problem

**DOI:** 10.12688/f1000research.2-54.v2

**Published:** 2013-08-07

**Authors:** Leonardo Valentin, Andrew DiNardo, Elizabeth Chiao, Laila Woc-Colburn, Arun Nachiappan

**Affiliations:** 1 Department of Radiology, Baylor College of Medicine, Houston, TX, 77030, USA; 2 Section of Infectious Diseases, Department of Medicine, Baylor College of Medicine, Houston, TX, 77030, USA

**Keywords:** Mycobacterium tuberculosis, Immune Reconstitution Inflammatory Syndrome, HAART

## Abstract

We present a case of a 39-year-old male patient with Acquired Immune Deficiency Syndrome (AIDS) who developed *Mycobacterium tuberculosis *related Immune Reconstitution Inflammatory Syndrome (IRIS) after initiation of Highly Active Antiretroviral Therapy (HAART) treatment. The inflammatory response resulted in mediastinal necrotic lymphadenopathy and subsequent perforation of the esophageal wall.

## Presentation

A 39-year-old man with a history of Acquired Immune Deficiency Syndrome (AIDS) presented to the emergency room with fever, productive cough, fatigue, diarrhea, and weight loss. Three weeks prior, he had been initiated on antiretroviral therapy (ART) with darunavir, ritonavir and combination tenofovir and emtricitabine. At that time, he had a CD4 count of 85 cells/μL (9%) and HIV-1 viral load of 336,950 RNA copies/mL. He was now febrile (41.0°C), with a heart rate of 100 beats/min and respiratory rate of 24/min. Physical examination revealed oral thrush and palpable cervical, supraclavicular and axillary lymphadenopathy. His laboratory evaluation was significant for a CD4 count of 28 cells/uL (10%), HIV-1 viral load of 3,410 RNA copies/mL, and hemoglobin of 6.6 g/dL. Chest radiograph on admission (not shown) demonstrated a 2.9 × 4.4 cm soft tissue mass in the anterior mediastinum.

**Figure 1.**
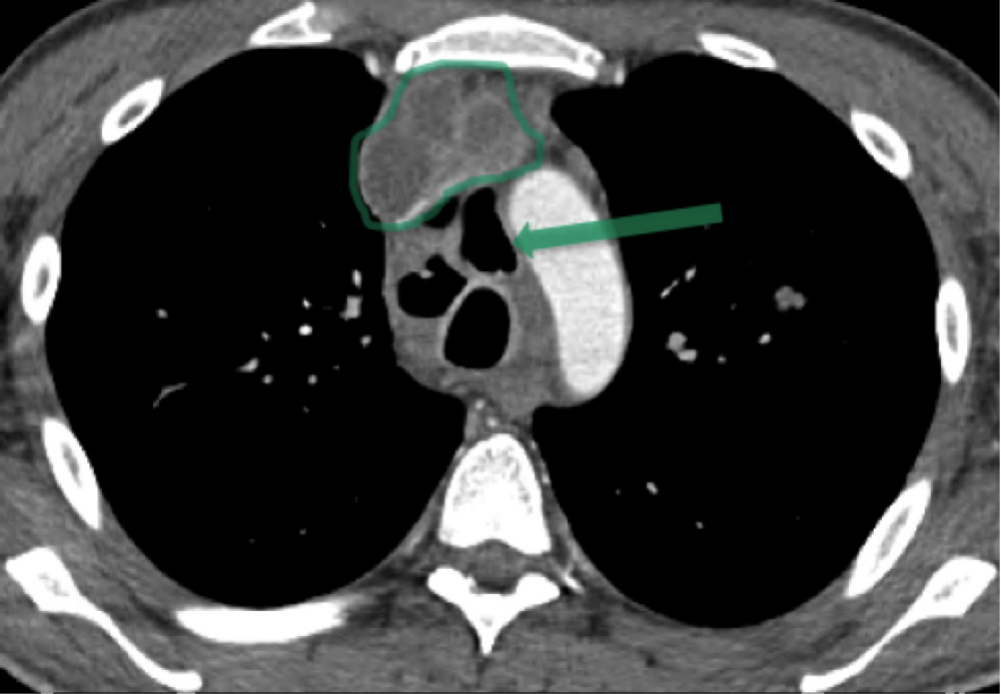
Contrast-enhanced CT scan of the chest on admission revealed multiple low-attenuation necrotic lymph nodes (free-hand-circle) and gas-containing mediastinal collection, representing a mediastinal abscess (arrow).

**Figure 2.**
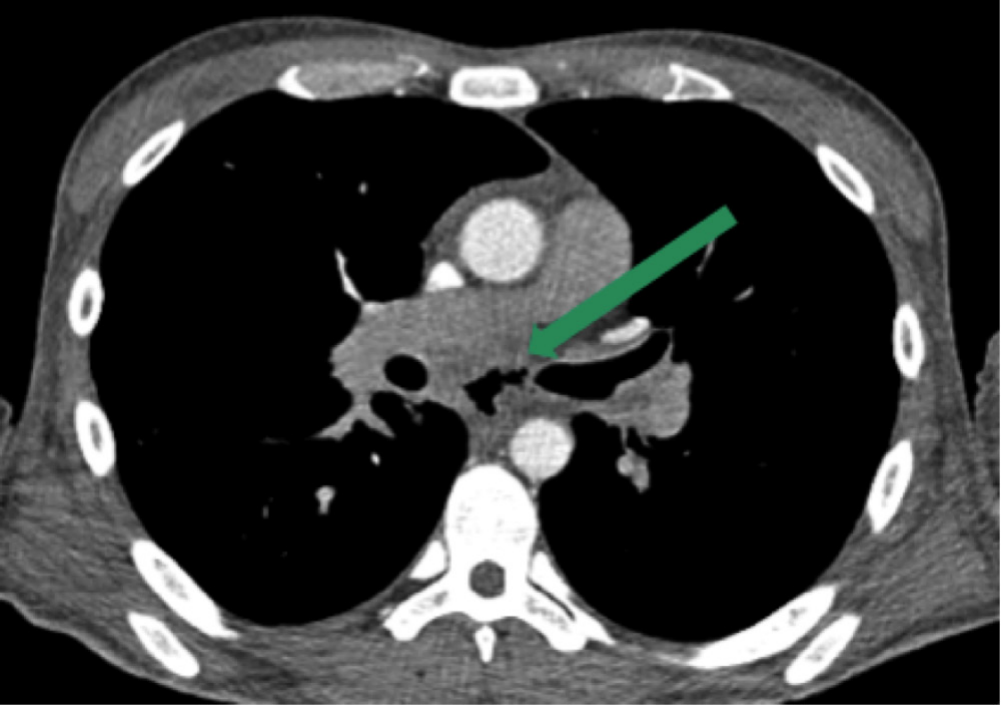
Chest CT on admission revealed esophageal discontinuity in the subcarinal region representing esophageal perforation (arrow).

**Figure 3.**
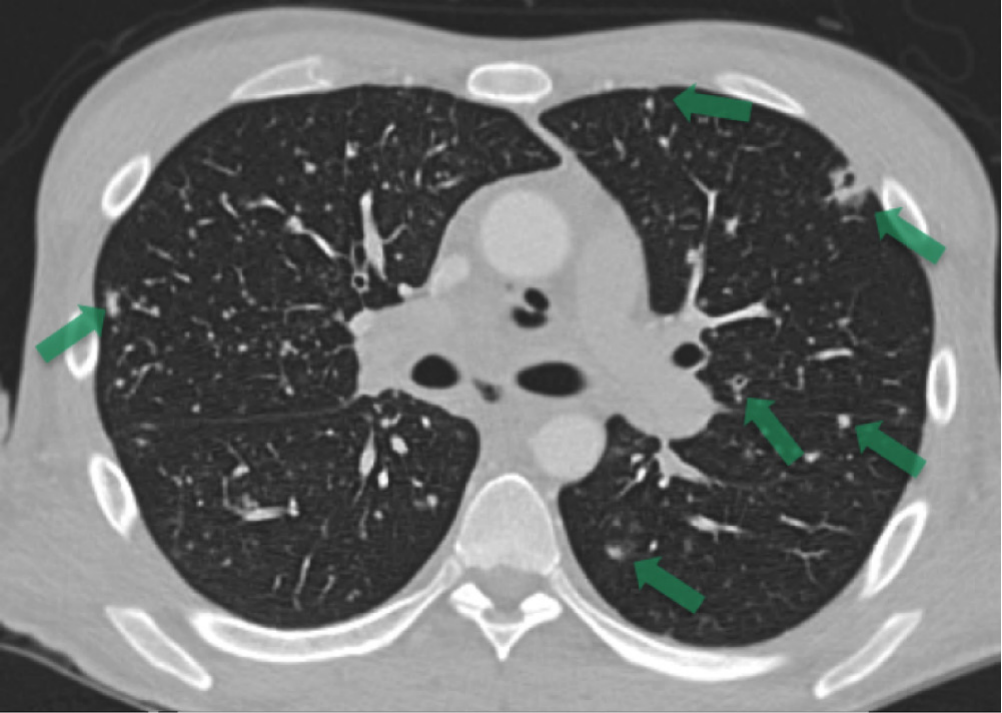
Chest CT on admission revealed multiple cavitary and non-cavitary lung nodules (arrows), suspicious for mycobacterial infection.

The initial computed tomography (CT) scan of the chest showed multiple low-attenuation mediastinal lesions, indicative of abscesses or necrotic lymphadenopathy ( Figure 1), as well as esophageal discontinuity in the subcarinal region, a sign of esophageal fistula or perforation ( Figure 2). Multiple cavitary lung nodules were also present ( Figure 3).

## Diagnosis

Mediastinoscopy revealed purulent fluid drainage from necrotic lymph nodes on day 3 of hospitalization. An esophagogastroduodenoscopy (EGD) demonstrated a 2 cm linear tear in the esophagus with proximal perforation at the 29–31 cm level.

The mediastinal fluid was found to be 4+ acid-fast bacilli (AFB) smear positive. PCR of the mediastinal fluid was also positive for *Mycobacterium tuberculosis* (MTB) complex on day 5 of hospitalization and the patient was started on IV linezolid, amikacin, rifampin and levofloxacin and transitioned to standard 4 drug anti-tuberculosis therapy (isoniazid, rifabutin, ethambutol and pyrazinamide) on day 8 when gastrointestinal access was obtained. Antiretroviral therapy had been held on day 2 and was subsequently resumed on day 9. The clinical presentation, recent initiation of ART, current 2-log decrease in viral load, and thoracic CT findings suggested a diagnosis of unmasking MTB immune reconstitution inflammatory syndrome (IRIS).

## Discussion

IRIS, previously known as immune restoration disease (IRD) and immune reconstitution syndrome (IRS) is a paradoxical deterioration in the clinical status of a patient after initiation of antiretroviral therapy ^1, 2^. The pathophysiology is related to the inflammation that occurs when the recovered immune system targets either live microorganisms or antigens from dead microorganisms ^3– 7^. Although recently proposed criteria for IRIS differ slightly, most criteria include the evidence of a recovered immune system along with a decrease in HIV viral load and/or increase in CD4 cell count. IRIS may occur as a paradoxical worsening of a known disease that has been under control with treatment, or an unmasking of a previously unsuspected disease ^4^. Common pathogens associated with IRIS include tuberculous and non-tuberculous mycobacteria, cytomegalovirus, *Pneumocystis jirovecii*, JC virus, *Cryptococcus neoformans*, herpes simplex virus, hepatitis B virus, hepatitis C virus and Kaposi sarcoma ^4, 5^. Non-infectious diseases such as sarcoidosis, Grave’s disease and thrombotic thrombocytopenic purpura have also been described, suggesting that IRIS is not only an exuberant reaction to live or non-viable organisms, but also a manifestation of an unbalanced immune system ^8^. While IRIS can occur acutely for up to 18 months after initiation of ART, most cases occur within the first two weeks to two months after initiation. IRIS is more likely to occur in the setting of high viral loads and low CD4 counts at the time of initiation of ART ^9– 11^.

In pre- and early Highly Active Antiretroviral Therapy (HAART) studies, the most common cause of lymphadenopathy (as seen on imaging) for an HIV patient with a CD4 count less than 50 cells/μL is mycobacterial infection ^12^. Determining the presence of IRIS is not always straightforward; however, several key features help support correct diagnosis. The most common imaging feature in MTB-IRIS includes lymph node enlargement with central necrosis, most commonly located in the abdominal, axillary and mediastinal distributions ^13^. The marked mediastinal lymphadenopathy in our patient is of particular interest, as this is common in patients with MTB-related IRIS ^14^.

This patient initiated ART with a low CD4 count of 85 cells/uL (9%) and a high HIV viral load of 336,950 RNA copies/mL. After initiation of ART, his viral load decreased by 2 logs to 3,410 RNA copies/mL. While the absolute CD4 count decreased, the percentage increased. The absolute CD4 decrease was likely related to mycobacterial bone marrow invasion and subsequent inflammation causing pancytopenia and total leukocytosis. Because of this common phenomenon, some authors question the efficacy of CD4 rise as part of a proposed standardized IRIS diagnostic criteria ^3^.

The exaggerated immune response to our patient’s mediastinal mycobacterial burden resulted in extension of inflammation from necrotic lymphadenopathy to the esophageal wall, which underwent necrosis and perforation. This resulted in a gas collection replacing the necrotic lymph nodes ( Figure 1). Esophageal perforation can occur from extensive coughing and retching in an MTB patient, with or without an underlying infectious esophagitis.

## Management

Although definitive management of IRIS has not been established by carefully controlled studies, current management may include the addition of corticosteroids and in severe cases, temporarily withholding ART. Case reports suggest non-steroidal anti-inflammatory drugs (NSAIDS) may offer symptomatic relief, however randomized evidence of this effect is lacking ^15^. Future management may include evaluation for a combination of cytokines and inflammatory markers such as interleukin 7, interleukin 6 and/or C-reactive protein to predict who is at higher risk of developing IRIS, which can be assessed prior to initiation of ART ^16, 17^. Future therapies may include immunomodulatory medications (C-C chemokine receptor 5 inhibitors, TNF antagonists or interleukin 6 receptor inhibitors) to temper the vigorous immune reconstitution.

**Figure 4.**
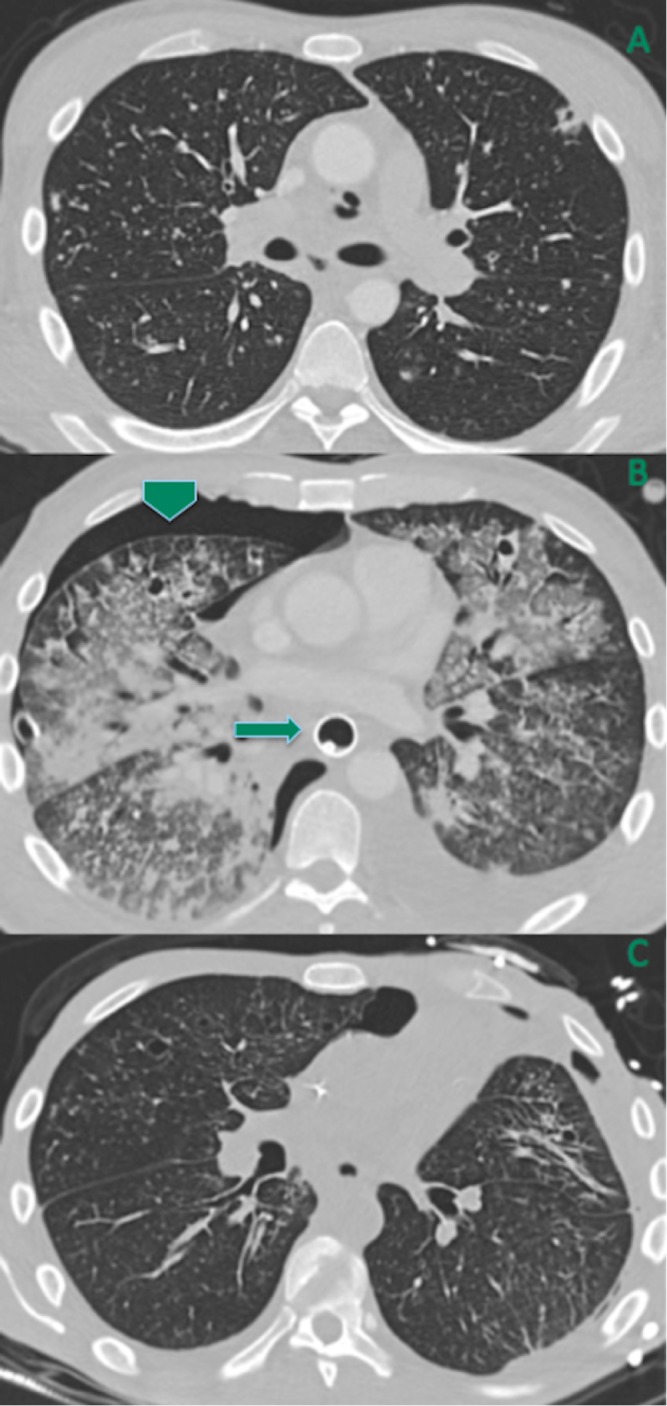
Sequential chest CT studies. ( **A**) Multiple cavitary and non-cavitary lung nodules (same as Figure 3). ( **B**) Hospital day 6: increased pulmonary tree-in-bud nodules and consolidations, new small right-sided pneumothorax (arrowhead), and new esophageal stent (arrow). ( **C**) One month follow-up: nearly-resolved pulmonary opacities decreased tiny right pneumothorax, and removal of esophageal stent.

Our patient had a complicated hospitalization including recurrent pneumothoraces ( Figure 4), empyema, and unmasking of cutaneous Kaposi sarcoma. Antiretroviral therapy was continued, except for a brief interruption between hospital days 2 and 9, throughout the hospitalization. As previously mentioned, antituberculous treatment was started after active *M. tuberculosis* infection was confirmed and the treatment regimen included isoniazid, rifabutin, ethambutol, and pyrazinamide.

The esophagus in a patient with a low CD4 count is vulnerable to infection ^18^. Our case illustrates a mediastinal infectious process in which TB-IRIS was the etiology and causative factor for an esophageal perforation that further complicated the treatment of this patient with AIDS.

## Consent

Written informed consent for publication of clinical details and clinical images was obtained from the patient.
