# Supplementary material for: Case Report: Tuberculosis IRIS: a mediastinal problem
Source: F1000Res. 2013 Aug 7;2:54. Originally published 2013 Feb 18. [Version 2] doi: 10.12688/f1000research.2-54.v2 (PMC3799548; doi:10.12688/f1000research.2-54.v2)
Supplement: Peer review history

# Peer review: Referee response for version 2

**DOI:** 10.5256/f1000research.1805.r3282

**Published:** 2014-01-23

**Authors:** Eric Daar

**Affiliations:** 1 David Geffen School of Medicine, University of California, Los Angeles, Los Angeles, CA, USA

I have read this submission. I believe that I have an appropriate level of expertise to confirm that it is of an acceptable scientific standard.

# Peer review: Referee response for version 2

**DOI:** 10.5256/f1000research.1805.r1526

**Published:** 2013-08-19

**Authors:** Susana Asin

**Affiliations:** 1 Department of Microbiology and Immunology, Dartmouth Medical School, Hanover, NH, USA

This revision has been significantly improved. The authors have addressed my comments and comments from additional reviewers

I have read this submission. I believe that I have an appropriate level of expertise to confirm that it is of an acceptable scientific standard.

# Peer review: Referee response for version 2

**DOI:** 10.5256/f1000research.1805.r1398

**Published:** 2013-08-09

**Authors:** Paul Klenerman

**Affiliations:** 1 Nuffield Department of Clinical Medicine, University of Oxford, Headington, Oxford, UK

I have read this submission. I believe that I have an appropriate level of expertise to confirm that it is of an acceptable scientific standard.

# Peer review: Referee response for version 1

**DOI:** 10.5256/f1000research.305.r998

**Published:** 2013-06-12

**Authors:** Paul Klenerman

**Affiliations:** 1 Nuffield Department of Clinical Medicine, University of Oxford, Headington, Oxford, UK

This is an interesting short case report and the images are striking.

A couple of points:

- The actual therapy for this patient was not described in any detail.
- The timing of therapy for TB/HIV disease could be discussed.
- The evidence for use of anti-inflammatory agents could be discussed and referenced.
- It would help to expand on the apparent drop in CD4+ T cell count and anemia. What were the other blood indices and how did these recover over time?
- As a small point the 1st line of abstract should be “39 year-old”.

I have read this submission. I believe that I have an appropriate level of expertise to confirm that it is of an acceptable scientific standard.

# Peer review: Referee response for version 1

**DOI:** 10.5256/f1000research.305.r953

**Published:** 2013-05-15

**Authors:** Susana Asin

**Affiliations:** 1 Department of Microbiology and Immunology, Dartmouth Medical School, Hanover, NH, USA

This is an interesting case report, about a mediastinal manifestation of unmasking Tuberculosis Immune Reconstitution Inflammatory Syndrome (IRIS). Specific remarks are:

1- Since the optimal time to start ARV in the course of anti-tuberculosis treatment is unclear detailed comments about case management i.e. TB-treatment are encouraged. 

2- The discussion should consider the fact that even though ARV decrease HIV-1 viral load, CD4 levels after three weeks of treatment were even lower (85 cells/ul compared to 28 cells/ul). Was this a clear indication of a recovering immune system? Should an increase in CD4 cells count have been expected?. 

3-This report underscores the relevance of an inflammatory response targeting either live microorganisms or antigens from dead microorganisms as the pathophysiology of IRIS. Is there any evidence to support ARVs as a contributing factor to the on-going inflammatory response?

I have read this submission. I believe that I have an appropriate level of expertise to confirm that it is of an acceptable scientific standard.

# Response

**Published:** 2013-06-16

**Authors:** Leonardo Valentin

**Affiliations:** Baylor College of Medicine, USA

Thank you for your review Dr. Asin. See below for our response:

- The patient had been inconsistently receiving anti-retroviral treatment since his diagnosis of HIV 10 years ago. Three weeks after having restarted antiretroviral therapy (ART) the patient presented to the emergency department with fever, productive cough, diarrhea, fatigue and weight loss. During his admission ART was continued, albeit with a brief interruption between hospital days 2 and 9, during his admission. Mediastinoscopy was performed on hospital day 3. The following day, PCR for TB was positive and the patient was started on four-drug anti-tuberculous therapy (ATT) consisting of Isoniazid, Rifabutin, Ethambutol, and Pyrazinamide. 
- While the absolute CD4 count decreased, the percentage increased. We believe the absolute CD4 decrease was likely related to mycobacterial bone marrow invasion and subsequent inflammation causing pancytopenia & total leukocytosis. In addition CD4 increases are known to lag behind viral load decrease and are relatively delayed to the IRIS event. Some authors question the efficacy of CD4 rise as part of a proposed standardized IRIS diagnostic criteria. 
*Reference:*
Achenbach, C. J. *et al.* 2012). Paradoxical immune reconstitution inflammatory syndrome in HIV-infected patients treated with combination antiretroviral therapy after AIDS-defining opportunistic infection. Clin Infect Dis. 54(3), 424–33. doi:10.1093/cid/cir8023.
  While the pathophysiology is unclear, the varied and multiple presentations of IRIS can provide some insight. There are probably some cases in which IRIS occurs due to neither dead nor live organisms, but an unbalanced immune system. Manifestations like thrombotic thrombocytopenic purpura, Grave’s thyroiditis, and other autoimmune conditions provide some examples of the non-infectious presentations.
*Reference:* 
Mounzer K, DiNardo A, Goldstein K: Thrombotic thrombocytopenic purpura during immune reconstitution.AIDS. (2007); 21: 2559–60.

# Peer review: Referee response for version 1

**DOI:** 10.5256/f1000research.305.r836

**Published:** 2013-03-14

**Authors:** Eric Daar

**Affiliations:** 1 David Geffen School of Medicine, University of California, Los Angeles, Los Angeles, CA, USA

This is an interesting case report of a somewhat novel manifestation of TB IRIS.  The comments related to IRIS are relevant and of some value.  Minor comments include:

- The discussion under management probably should at least raise the importance of  continuing pathogen specific therapy and the potential role of nonsteroidal anti-inflammatory agents.
- Page 3, bottom of column 1, top of column 2 states “The esophagus in a HIV patient is particularly vulnerable to pathology.”   The use of the term “HIV Patient” is awkward and the overall statement is not supported by any data or citation.

I have read this submission. I believe that I have an appropriate level of expertise to confirm that it is of an acceptable scientific standard.

# Response

**Published:** 2013-06-13

**Authors:** Leonardo Valentin

**Affiliations:** Baylor College of Medicine, USA

Thank you for your report Dr. Daar. Please find below the response to some of the comments: 

- Case reports suggest NSAIDS may offer symptomatic relief, however randomized evidence for this is lacking. There is a BIII recommendation to use NSAIDS for TB- associated IRIS according to the updated IDSA/CDC guidelines.
*References:*
Murdoch DM. Incidence and risk factors for the immune reconstitution inflammatory syndrome in HIV patients in South Africa: a prospective study. AIDS. 2008;22(5):601
Centers for Disease Control and Prevention. Guidelines for Prevention and Treatment of Opportunistic Infections in HIV-Infected Adults and Adolescents. MMWR 2009;58 (No. RR-4) April 10, 2009. Updated March 2013.
- Patients with very low CD4 counts commonly have esophageal pathology, most commonly due to *Candida*, CMV or HSV and more rarely due to HPV, mycobacteria, idiopathic ulceration, syphilis or *H. ducreyi.*
*Reference:*
Wilcox CM. Esophageal disease in acquired immunodeficiency syndrome: etiology, diagnosis, and management. Am J Med 1992 Apr 92 412-421.
